# Supplementary material for: Macroscopic control of cell electrophysiology through ion channel expression
Source: eLife. 2022 Nov 9;11:e78075. doi: 10.7554/eLife.78075 (PMC9711524; doi:10.7554/eLife.78075)
Supplement: Supplementary file 1. [file elife-78075-supp1.docx]

**Supplementary file 1.**

Oligos and synthetic DNA sequences used in this study.

| **Oligo/synthetic DNA name** | **sequence** |
| --- | --- |
| VECGAD_R | gccttcatgctccttgatttcc |
| GAD_F | tcttcatgaaaatatattacgagggct |
| TCYC_F | CCGCATCATGTAATTAGTTATGTCACG |
| HIS_F | CCGGGCGAATTTCTTATGATTTATGAT |
| PMIN_R | TTAGTGTGTGTATTTGTATTTGCGTGTC |
| PMIN_F | GACACGCAAATACAAATACACACACTAA |
| IacR-VP64 | GACACGCAAATACAAATACACACACTAAATGTTGGACCACTTGGAACAATTCTTGCCAAACAAGGAACCATCTTCTATCCAAAACTTCCCATTCTTCTGGATCTCTCAAGTTAACGGTAAGTACTCTCAATTGATCGAAAAGTCTATCAAGAAGTTGGGTATCGACAACACTAGAAGAAAGATCATCTTGTCTACTAACGCTTTGGGTGAAGCTTCTATCACTGACATCGCTAACTTGTCTACTTTGAAGTTGACTACTGCTACTAAGGCTGTTTACAGATTGGTTGAAGACGGTATCGTTGAAGTTTACTCTTCTACTACTGACGAAAGAATCTCTATGGTTAAGTTGACTGCTAAGGGTGTTGAATTGGTTGAACAAATCAACCAAATCTCTGTTGTTACTTTGGCTGGTATCTTGAACGCTTTCTCTGAAGACGAATTGCACAACTTGAACCACCAATTGAAGAAGTTGTTCGACTTGATGCCATCTTCTagcagggctgacggttctggtagatctggagtcgacggtggaggttctGACGCATTGGACGATTTTGATCTGGATATGCTGGGAAGTGACGCCCTCGATGATTTTGACCTTGACATGCTTGGTTCGGATGCCCTTGATGACTTTGACCTCGACATGCTCGGCAGTGACGCCCTTGATGATTTCGACCTGGACATGCTGATTAACggcagcCCCAAGAAGAAGAGGAAAGTCGGGagtCAAAGCCATGGTTTTCCGCCTGAGGTGGAAGAACAAGATGATGGTACGCTGCCAATGTCATGTGCACAAGAATCCGGGATGGATAGACACCCTGCCGCCTGTGCAAGTGCTCGTATCAACGTGTAAtcttcatgaaaatatattacgagggct |
| MarR-RD | GACACGCAAATACAAATACACACACTAAATGAAGTCTACTTCTGACTTGTTCAACGAAATCATCCCATTGGGTAGATTGATCCACATGGTTAACCAAAAGAAGGACAGATTGTTGAACGAATACTTGTCTCCATTGGACATCACTGCTGCTCAATTCAAGGTTTTGTGTTCTATCAGATGTGCTGCTTGTATCACTCCAGTTGAATTGAAGAAGGTTTTGTCTGTTGACTTGGGTGCTTTGACTAGAATGTTGGACAGATTGGTTTGTAAGGGTTGGGTTGAAAGATTGCCAAACCCAAACGACAAGAGAGGTGTTTTGGTTAAGTTGACTACTGGTGGTGCTGCTATCTGTGAACAATGTCACCAATTGGTTGGTCAAGACTTGCACCAAGAATTGACTAAGAACTTGACTGCTGACGAAGTTGCTACTTTGGAATACTTGTTGAAGAAGGTTTTGCCAagcagggctgacggttctggtagatctggagtcgacggtggaggttctGACGCATTGGACGATTTTGATCTGGATATGCTGGGAAGTGACGCCCTCGATGATTTTGACCTTGACATGCTTGGTTCGGATGCCCTTGATGACTTTGACCTCGACATGCTCGGCAGTGACGCCCTTGATGATTTCGACCTGGACATGCTGATTAACggcagcCCCAAGAAGAAGAGGAAAGTCGGGagtCAAAGCCATGGTTTTCCGCCTGAGGTGGAAGAACAAGATGATGGTACGCTGCCAATGTCATGTGCACAAGAATCCGGGATGGATAGACACCCTGCCGCCTGTGCAAGTGCTCGTATCAACGTGTAAtcttcatgaaaatatattacgagggct |
| dEGFP(UBG76V-EGFP) | GACACGCAAATACAAATACACACACTAAatgcagatcttcgttaaaacattgaccgggaaaaccatcactctagaggttgaaccatcagacacgatcgagaatgttaaagctaaaattcaagacaaggaaggtattcctccagaccaacagcgtttaatcttcgctggtaaacaattggaagatggcagaacacttagcgattataacatccagaaagaaagtacattgcatttagtgcttcgactcaggggtgttgttggtaaactcggaagacaggatccgcctgtagcaacaATGTCTAAAGGTGAAGAATTATTCACTGGTGTTGTCCCAATTTTGGTTGAATTAGATGGTGATGTTAATGGTCACAAATTTTCTGTCTCCGGTGAAGGTGAAGGTGATGCTACTTACGGTAAATTGACCTTAAAATTTATTTGTACTACTGGTAAATTGCCAGTTCCATGGCCAACCTTAGTCACTACTTTCGGTTATGGTGTTCAATGTTTTGCTAGATACCCAGATCATATGAAACAACATGACTTTTTCAAGTCTGCCATGCCAGAAGGTTATGTTCAAGAAAGAACTATTTTTTTCAAAGATGACGGTAACTACAAGACCAGAGCTGAAGTCAAGTTTGAAGGTGATACCTTAGTTAATAGAATCGAATTAAAAGGTATTGATTTTAAAGAAGATGGTAACATTTTAGGTCACAAATTGGAATACAACTATAACTCTCACAATGTTTACATCATGGCTGACAAACAAAAGAATGGTATCAAAGTTAACTTCAAAATTAGACACAACATTGAAGATGGTTCTGTTCAATTAGCTGACCATTATCAACAAAATACTCCAATTGGTGATGGTCCAGTCTTGTTACCAGACAACCATTACTTATCCACTCAATCTGCCTTATCCAAAGATCCAAACGAAAAGAGAGACCACATGGTCTTGTTAGAATTTGTTACTGCTGCTGGTATTACCCATGGTATGGATGAATTGTACAAAggcagcCCCAAGAAGAAGAGGAAAGTCGGGagtTAACCGGGCGAATTTCTTATGATTTATGAT |
| pMarIac | ggaaatcaaggagcatgaaggcagctaagcaataacttagtaaaaaaagggtaattctattataaacaaaattttgcatatttccttgaacgcacattagaaaaattacttgacaattccattaaattttctgatattttaattaatggataaaaatacatggaaaagtgaCATTACTCGCATCCATTCTCAGGCTGACTTATACTTGCCTGGGCAATATTATCCCATGCAACTAATTACTTGCCAGGGCAACTAATGTGGCATATATATATGTGCGCGTATATACATGATTATATGGCATGTATGTGCTCTGTATGTATATAAAACTCTTTTTTTCTTTTTTTCTCTAAATTTTTTTTCCTTATACATTAGGACCTTTGCAGCATAAATTACTATACTTCTATAGACACGCAAATACAAATACACACACTAA |
| KcsA* | GACACGCAAATACAAATACACACACTAAATGCCCCCGATGCTGAGTGGTTTACTGGCTAGGCTGGTTAAACTTTTATTAGGCAGACATGGCAGTGCGCTTCAATGGAGAGCTGCCGGTGCTGCCACCGTCTTACTTGTTATAGTATTGCTGGCCGGTAGTTATCTAGCCGTCCTTGCGGAGAGAGGCGCTCCTGGTGCTCAGCTTATCACATATCCCCGTGCTCTGTGGTGGAGCGTTGAAACCGCGACTACAGTTGGCTATGGAGACTTGTACCCTGTGACATTGTGGGGCCGTTTGGTAGCAGTTGTAGTAATGGTGGCAGGCATAACTAGCTTTGGCCTAGTTACTGCAGCCTTAGCAACGTGGTTCGTGGGACAGGAGCAACAACAGCAGGGGCAGTTTGTGAGGCATTCCGAGAAAGCAGCCGAGGAGGCTTACACCAGAACTACGCGTGCGCTTCACGAGAGATTTGACAGACTGGAACGTATGCTGGATGACAATAGACGTGCCGCCGCGAAATCTCGTATAACCTCTGAGGGGGAATACATTCCACTGGATCAAATCGACATCAATGTAGAACAGGATGATGGGACTCTACCTATGTCATGTGCCCAAGAGAGCGGCATGGATAGGCATCCAGCAGCATGTTTCTGTTACGAAAATGAAGTCTAACCGGGCGAATTTCTTATGATTTATGAT |

| TOK1* | GACACGCAAATACAAATACACACACTAAatgacaaggttcatgaacagctttgccaaacaaacgctgggatatggcaatatggcgacagtggagcaagagagctcagctcaggctgttgattctcattcaaacaacacaccgaagcaagctaagggtgttcttgcagaggaactaaaggatgcattgcggttccgggacgaaagagttagtattattaatgcagagccttcttcaacactgttcgtcttttggtttgtggtttcatgctatttccctgtgattactgcctgcttgggtcccgtagctaacactatctcgatagcctgtgtagttgaaaaatggagatccttaaagaacaactccgtggtgacaaatccacgaagcaatgacaccgatgttttgatgaatcaagtaaagacagtttttgatcctcctggtatttttgccgttaatatcatctctttggtactgggttttacgtcaaatattatactaatgctacatttcagtaagaagttgacgtatcttaaatctcagttaataaatataacaggatggacaatagctggagggatgcttttggtggacgtgattgtatgctccttgaatgacatgcccagcatctacagtaagactatcggattttggtttgcctgtatcagttctggtctatatttggtatgcaccattattttaacaatacattttattggatataaattaggaaaatatcctccaacgttcaaccttttgcccaatgaaagaagtatcatggcatacactgtactattgtctttatggttgatttggggtgcgggtatgtttagcggtttattgcacatcacttacggaaatgcattatatttctgcacggtatcattattaaccgtgggactaggtgacatcctgcccaagtcggttggcgccaaaatcatggttttaatcttttcgctatctggtgttgtcttgatgggtttaatagtgtttatgacaagatccatcattcaaaagtcctctggcccaattttctttttccacagagttgaaaaaggcaggtccaaatcgtggaaacattatatggatagtagtaaaaatttatctgaaagggaagcgttcgacttaatgaagtgtatccgacaaacggcctcaaggaagcagcattggttttctttgtcggtgactattgcaattttcatggctttttggttattgggagctcttgtattcaaattcgcagaaaattggtcgtacttcaattgtatttacttttgtttcttgtgcttattaaccattggatacggagactatgctccaaggactggtgcaggccgtgctttttttgtgatttgggcgttgggagccgtgccattaatgggggctatcctatctacagtcggtgatctgttgtttgacatttccacttctctggatattaagatcggtgaatcattcaataataaagtcaagtccatcgtttttaatgggcgtcaaagagcactttcctttatggtgaacactggagaaattttcgaagaatctgacacagctgatggtgatctggaagaaaatacaacgagctcacaatccagtcaaatttctgaattcaacgataataattcagaagagaatgattctggagtgacatcccctcctgcaagcctgcaagaatcattttcttcattatcaaaagcatctagcccagagggaatacttcctctagaatatgtttcttctgctgaatatgcactacaggactcggggacctgtaatttaaggaacttgcaagagctacttaaagccgtcaaaaaactacatcggatatgtctggcggataaagattacacacttagtttttccgactggtcgtacattcataaactacatttgaggaacattacagatattgaggagtacacacgcggacccgaattttggatatcacctgatacgcccctcaagttcccgttaaatgaacctcattttgcttttatgatgcttttcaagaacatagaagaattagttggtaatctagtagaagacgaagagctttataaagttataagcaaaagaaaatttttgggtgagcatagaaagacacttGCGTGCAAAAACTGGTTTTCCTCCTTAAGTCACTTCGTAATTCATTTATAACCGGGCGAATTTCTTATGATTTATGAT |
| --- | --- |
| KcsA-EGFP | GACACGCAAATACAAATACACACACTAAATGCCCCCGATGCTGAGTGGTTTACTGGCTAGGCTGGTTAAACTTTTATTAGGCAGACATGGCAGTGCGCTTCAATGGAGAGCTGCCGGTGCTGCCACCGTCTTACTTGTTATAGTATTGCTGGCCGGTAGTTATCTAGCCGTCCTTGCGGAGAGAGGCGCTCCTGGTGCTCAGCTTATCACATATCCCCGTGCTCTGTGGTGGAGCGTTGAAACCGCGACTACAGTTGGCTATGGAGACTTGTACCCTGTGACATTGTGGGGCCGTTTGGTAGCAGTTGTAGTAATGGTGGCAGGCATAACTAGCTTTGGCCTAGTTACTGCAGCCTTAGCAACGTGGTTCGTGGGACAGGAGCAACAACAGCAGGGGCAGTTTGTGAGGCATTCCGAGAAAGCAGCCGAGGAGGCTTACACCAGAACTACGCGTGCGCTTCACGAGAGATTTGACAGACTGGAACGTATGCTGGATGACAATAGACGTGCCGCCGCGAAATCTCGTATAACCTCTGAGGGGGAATACATTCCACTGGATCAAATCGACATCAATGTAGGCTCCATGTCTAAAGGTGAAGAATTATTCACTGGTGTTGTCCCAATTTTGGTTGAATTAGATGGTGATGTTAATGGTCACAAATTTTCTGTCTCCGGTGAAGGTGAAGGTGATGCTACTTACGGTAAATTGACCTTAAAATTTATTTGTACTACTGGTAAATTGCCAGTTCCATGGCCAACCTTAGTCACTACTTTCGGTTATGGTGTTCAATGTTTTGCTAGATACCCAGATCATATGAAACAACATGACTTTTTCAAGTCTGCCATGCCAGAAGGTTATGTTCAAGAAAGAACTATTTTTTTCAAAGATGACGGTAACTACAAGACCAGAGCTGAAGTCAAGTTTGAAGGTGATACCTTAGTTAATAGAATCGAATTAAAAGGTATTGATTTTAAAGAAGATGGTAACATTTTAGGTCACAAATTGGAATACAACTATAACTCTCACAATGTTTACATCATGGCTGACAAACAAAAGAATGGTATCAAAGTTAACTTCAAAATTAGACACAACATTGAAGATGGTTCTGTTCAATTAGCTGACCATTATCAACAAAATACTCCAATTGGTGATGGTCCAGTCTTGTTACCAGACAACCATTACTTATCCACTCAATCTGCCTTATCCAAAGATCCAAACGAAAAGAGAGACCACATGGTCTTGTTAGAATTTGTTACTGCTGCTGGTATTACCCATGGTATGGATGAATTGTACAAAggcagcGAACAGGATGATGGGACTCTACCTATGTCATGTGCCCAAGAGAGCGGCATGGATAGGCATCCAGCAGCATGTTTCTGTTACGAAAATGAAGTCTAACCGGGCGAATTTCTTATGATTTATGAT |
