# Supplementary material for: Macroscopic control of cell electrophysiology through ion channel expression
Source: eLife. 2022 Nov 9;11:e78075. doi: 10.7554/eLife.78075 (PMC9711524; doi:10.7554/eLife.78075)
Supplement: Supplementary file 2. [file elife-78075-supp2.docx]

**Supplementary file 2.** Model parameters.

| **Model parameter** | **Value** |
| --- | --- |
| $a_{1}$ | 0.15 |
| $a_{2}$ | 0.05 |
| $b_{1}$ | 7.5 |
| $b_{2}$ | 10 |
| $K_{A}$ | 5000 |
| $K_{B}$ | 75000 |
| $d_{1}$ | 0.0000386(Figure 2 – figure supplement 1A and Figure 2D); 0.000386(Figure 2 – figure supplement 1B and 1C and Figure 2B and 2D) |
| $d_{2}$ | 0.0000386(Figure 2 – figure supplement 1A, B and Figure 2B and 2D);  0.0000965 (Figure 2 – figure supplement 1C) |
| $\epsilon_{I}$ | 0 (Figure 2 – figure supplement 1A-C); otherwise, 10 |
| $\epsilon_{M}$ | 0 (Figure 2 – figure supplement 1A-C); otherwise, 20 |
